# The neural representation of mental beliefs held by two agents

**DOI:** 10.3758/s13415-019-00714-2

**Published:** 2019-04-12

**Authors:** Ceylan Özdem, Marcel Brass, Arjen Schippers, Laurens Van der Cruyssen, Frank Van Overwalle

**Affiliations:** 1 Department of Psychology, Vrije Universiteit Brussel, Pleinlaan 2, B - 1050 Brussels, Belgium; 2 University of Ghent & Ghent Institute for Functional and Metabolic Imaging, Ghent, Belgium

**Keywords:** Theory of Mind, Social mentalizing, Perspective taking, Temporo-parietal junction

## Abstract

Neuroimaging research has demonstrated that mentalizing about false beliefs held by other people recruits the temporo-parietal junction (TPJ). However, earlier work was limited to a single agent that held a false belief. We investigated the effect of two agents that held similar or mixed false and/or true beliefs. Participants saw animated stories with two smurfs holding true or false beliefs (Story phase). At the end of each trial, they were requested to take the perspective of the self or one of the smurfs (Question phase). We predicted that an increasing number of smurfs holding a false belief would increase activation in the TPJ when participants have to report the belief of the smurf, because the incongruent belief should have a stronger influence if it is held by two compared with one agent. This prediction was confirmed as activation in the TPJ during the Story and Question phase increased when more smurfs held a false belief. Taking the perspective of the self led to stronger activation of the TPJ in the two conditions that involved a true belief and weakest activation in the condition of two false beliefs. These data suggest that activation in TPJ depends on the perspective participants take, and that the number of agents holding a false belief influences activation in the TPJ only when taking the agent’s perspective.

**Electronic supplementary material:**

The online version of this article (10.3758/s13415-019-00714-2) contains supplementary material, which is available to authorized users.

## Introduction

In our daily life, we often interact with other people. We usually make inferences about these people’s beliefs and mental states by observing their behaviors or by reading their statements on social media posts. The ability to infer others’ mental states facilitates our social interaction and cooperation. This process is termed mentalizing or Theory of Mind (Premack & Woodruff, 1978). Research so far has focused on how we understand the mind of a single person (Wellman, Cross, & Watson, 2001), although in real conversations or chat sessions we often communicate with several people at the time. The purpose of this article is to investigate systematically how we attribute beliefs to multiple compared with single agents.

One of the main tasks that has been used to measure mentalizing capacity is the false belief task (Baron-Cohen, Leslie, & Frith, 1985). In this task, an object is displaced unbeknownst to an agent who is temporarily absent. The participant must infer the agent’s belief on where the object was previously, and because this belief conflicts with current reality, it is termed “false.” In their seminal work, Saxe and Kanwisher (2003) proposed that the temporo-parietal junction (TPJ) may serve to compute other’s beliefs and is involved in “reasoning about the content of mental states” (p. 1835). Neuroimaging research has since then confirmed that the TPJ is recruited when people select another’s perspective and belief (see meta-analyses by Schurz, Radua, Aichhorn, Richlan, & Perner, 2014; Van Overwalle, 2009; Van Overwalle & Baetens, 2009). In addition, the TPJ has been related to self-other distinction in the control of imitation, agency processing, and perspective taking (Brass, Ruby, & Spengler, 2009; Farrer & Frith, 2002; Ruby & Decety, 2001; Saxe & Powell, 2006; Saxe & Wexler, 2005), as well as in other nonsocial processes, such as spatial attention reorientation (Cabeza, Ciaramelli, & Moscovitch, 2012; Corbetta & Shulman, 2002; Krall et al., 2014; Mitchell, 2008; Özdem, Wiese, et al., 2017; Özdem, Brass, Van der Cruyssen, & Van Overwalle, 2017). Together, the TPJ plays a crucial role in social situations where we have to infer other people’s mental states or distinguish our own from another’s person perspective.

An important limitation of previous neuroimaging research on mentalizing and false beliefs is that it is restricted to the situations where false beliefs had to be inferred consistently for one agent only, either alone or in the presence of other agents. For instance, in pictorial stories (Bardi, Desmet, Nijhof, Wiersema, & Brass, 2017; Özdem, Brass, et al., 2017; Schuwerk et al., 2014), typically there is only one agent present who is unaware of the displacement and holds a false belief. On the other hand, in verbal stories (Saxe & Kanwisher, 2003), typically two agents are described of which one is aware that an object is displaced, while the other is unaware of this and his or her false belief is requested. These situations capture a variety of social situations where observers infer mental states of single and multiple agents. This raises at least two questions that have not yet been investigated systematically in the false belief literature. First, does it matter whether one or more agents hold a specific mental state? Second, what happens if two agents hold different mental states (one agent the same true belief as we do and the other a false belief)?

We therefore extended the classical false belief paradigm, and manipulated systematically whether two agents held similar or opposing true and/or false beliefs. To make the attribution of beliefs to the two agents an even more indispensable requirement for solving the task, participants did not know in advance whether they had to take the perspective of one of these agent’s or of their own, until after the belief formation was finished. Consequently, during the story phase, participants had to keep track of the presence of all agents involved or of their beliefs. A similar procedure was previously introduced in an fMRI study by Schuwerk, Döhnel. and colleagues (2014).

**Fig. 1.**
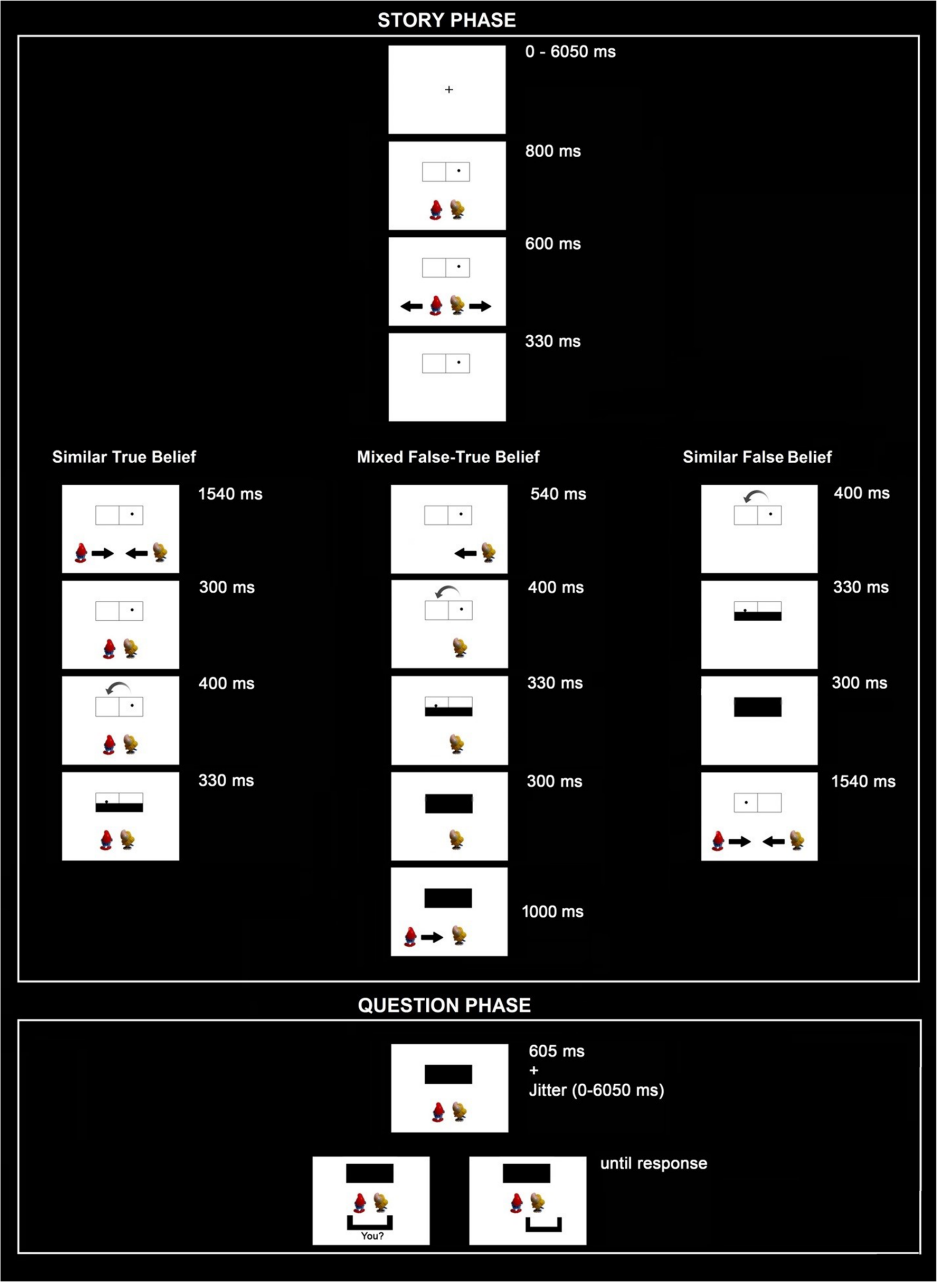
Stimuli and design of the belief task. Similar True beliefs are shown on the left, Mixed beliefs in the middle, and Similar False beliefs on the right. Perspective questions for the self (i.e., “you?”) and a smurf are shown at the end of each trial, but only one perspective was actually asked per trial

In the present experiment, we used an animated, spatial version of a false belief task adapted from Özdem, Brass, et al. (2017) (Fig. 1), in which two smurfs saw a black target circle that either stayed on the same side of a frame or jumped to the other side. Importantly, the circle jumped and afterwards the whole frame was covered, while the smurf was either present (resulting in a true belief) or absent (resulting in a false belief). Consequently, the two smurfs held either similar true beliefs, similar false beliefs, or mixed false-true beliefs (Table 1).

**Table 1.** Experimental conditions and predictions for the target Smurf perspective

Belief condition	Self	Target Smurf	Other Smurf
Similar true	True	True	True
Mixed true	True	True *	False
Mixed false	True	False **	True
Similar false	True	False ***	False

The Belief Conditions are named after the belief held by the Target Smurf and the Other Smurf respectively. The level of predicted activation from the smurf perspective is indicated with asterisks, and becomes stronger the more the smurfs hold false beliefs

Our main prediction is that when two smurfs hold false beliefs, this should lead to stronger processing in the mentalizing network than when only one smurf holds a false belief or none of them (i.e., both smurfs hold a true belief), and this should show up through slower behavioral responses and higher fMRI activation, particularly in the TPJ. Theoretically, the activation of the TPJ during false belief reasoning is assumed to depend on a process of “decoupling” away from one’s self-perspective on reality (Hartwright, Apperly, & Hansen, 2015; van der Meer, Groenewold, Nolen, Pijnenborg, & Aleman, 2011), also conceived as a process of “reorientation” from the current reality to one’s mental representation of the other smurfs’ knowledge and belief (Cabeza et al., 2012). This process of decoupling is expected to be more strongly recruited when two rather than one smurf may hold a false belief, leading to higher activation of the TPJ. Decoupling is expected to be absent when there are no false beliefs, leading to weaker TPJ activation (cf. meta-analysis by Krall et al., 2014). In contrast, we do not have strong predictions in the question phase when participants have to tell their own belief.

Recent work has demonstrated that decoupling away from one’s self-perspective on reality, also termed “self-inhibition,” is especially triggered when one’s self-perspective is made salient (Hartwright et al., 2015; van der Meer et al., 2011). Salience is high in the current study, because participants have to select constantly between either their own or the smurf’s perspective. This decoupling process increases executive control to monitor and detect potential conflicts in information input in the posterior medial frontal cortex (pmFC) (Botvinick, Cohen, & Carter, 2004), thereby triggering compensatory adjustments in cognitive control in the bilateral inferior frontal gyri (IFG) to avoid or override incorrect responses. In false belief reasoning, this entails overriding one’s prepotent true self-perspective to focus on the smurf’s false perspective (Hartwright et al., 2015; Hartwright, Apperly, & Hansen, 2012; Rothmayr et al., 2011; Schuwerk et al., 2014; van der Meer et al., 2011). For this reason, we predict a parallel pattern of activation in the pmFC as in the TPJ. Specifically, a gradual increase of activation when false beliefs are held by more smurfs. Also in parallel with the TPJ, we do not have strong predictions when a self-perspective is taken.

## Method

### Participants

Twenty-eight adults took part in this study (17 women; age range: 18-28 years; mean age: 22.1 years), and data of 25 participants were analyzed: three participants were excluded due to excessive head movements and one participant was excluded due to high error rates (above 50%). All participants were right-handed, as assessed by the Dutch version of the Edinburgh Inventory (Oldfield, 1971). They were paid 20 Euros for their participation. Participants reported no abnormal neurological history and had normal or corrected-to-normal vision. Participants gave informed consent before the experiment in accordance with the guidelines of the Medical Ethics Committee at the Ghent University Hospital (where scanning took place) and Brussels University Hospital (of the principal investigator FVO).

### Stimuli

We adapted the belief task and clips from a previous fMRI study by Özdem, Brass et al. (2017). Participants were presented with animated video clips. The animations consisted of a black ball moving in a frame subdivided in two adjacent (right-left) rectangular parts and two agents: Papa Smurf and Smurfette (Fig. 1). The location of the ball and the smurfs was counterbalanced in all tasks and conditions.

All trials in this task started with the frame, the ball presented either on the right or left rectangular part of this frame, and the smurfs underneath the frame. Next, the smurfs simultaneously left the scene towards the opposite sides of the screen that were closest to each of them (e.g., Papa Smurf to the right; Smurfette to the left). The side where the smurfs were initially positioned was counterbalanced across conditions. Next, the black ball jumped to the other side (left-right) of the frame followed by an occluder covering the frame. Depending on the condition, this happened in the presence or absence of one or both smurfs, that is, after the smurfs’ return to the original location so that it could witness the black ball jumping to the other location (i.e., true belief) or before the smurfs’ return so that the change was not witnessed (false belief).

After the occlusion, participants were asked to indicate as soon as possible where the ball was located (at the left or right part of the frame) according to Papa Smurf, Smurfette or themselves. Participants did not know which perspective they had to take until after the belief formation was finished to make the attribution of beliefs to the two agents an indispensable requirement for solving the task. The target agent of the question was presented by a black bar that appeared under one of the smurfs (implying the view according to that smurf) or with the word ”you?“ (in Dutch: “jou?”) in the middle of the screen to imply participant’s own perspective (Fig. 1). Participants had to answer as quickly as possible by pressing the corresponding left and right button. Participants were instructed to keep their eyes focused on the center of the frame at all times. Note that participants did not know who (self or smurf) would be the target of the question until the end of the trial.

### Design and procedure

The design of the experiment involved the within-participant factors Belief Type (true vs. false), Perspective (self vs. smurf), and Belief Content (similar vs. mixed). The conditions differed on the following aspects. First, a true versus false Belief Type actually refers to smurfs’ belief only, because participants always held a true belief as they witnessed any change in the location of the ball. Second, in the similar Belief Content condition, the two smurfs held the same beliefs, because they both returned to their initial position early, and they were able to observe the ball changing its location (similar true belief) or they both returned after the occluder went up so that they did not witness any change in the scene (similar false belief). In the mixed Belief Content condition, the two smurfs had divergent beliefs about the location of the ball, because one smurf returned early and observed the change in location (mixed true belief) while the other smurf returned late and did not witness it (mixed false belief). Note that in the initial story phase, these latter two conditions cannot be distinguished because the target smurf is not yet selected. Rather the conditions can only be distinguished in the question phase when the belief of either the smurf holding the true belief or the smurf holding the false belief had to be indicated.

In addition to these conditions, we had filler trials where the ball did not jump at all and stayed in the same location. The filler trials were used to prevent the expectation that the black ball would always jump to the other side, and hence forced the participants to pay attention to the whole task. The rest of the filler videos were varied in the same way as in the other experimental conditions. The belief task consisted of 182 trials in total with 24 trials in each experimental condition (96 trials in the similar condition with an equal number of true and false belief trials, 72 trials in the mixed condition with an equal number of true and false belief trials), and 14 fillers. In half of the conditions, participants were asked to take their own perspective, whereas in the other half, the participants were asked to take one of the smurf’s perspective.

Before scanning, participants received instructions and a practice session to ensure that they understood the instructions. All participants were assigned to a belief and memory task in the scanner in this order. All trials in the belief task were preceded by a jitter with variable duration between 0–6,050 ms and another jitter with variable duration between 0–6,050 ms was presented at the end of the video (Fig. 1). After the jitter, the question bar appeared and remained on the screen until the participant responded. The memory task was further ignored, because it did not yield significant activations and always came after the belief task.

### fMRI data acquisition

Images were obtained using a 3 T Magnetom Trio MRI scanner system (Siemens Medical Systems, Erlangen, Germany), using a 32-channel radiofrequency head coil. First, a high-resolution anatomical images were collected using a T1-weighted 3D MPRAGE sequence [TR = 2,530 ms, TE = 2.58 ms, TI = 1,100 ms, acquisition matrix = 256 × 256 × 176, sagittal FOV = 220 mm, flip angle = 7, voxel size = 0.9 × 0.86 × 0.86 mm3 (resized to 1 × 1 × 1 mm^3^)]. Second, a fieldmap was calculated to correct for inhomogeneities in the magnetic field (Cusack & Papadakis, 2002). Next, whole brain functional images were acquired by using a T2*-weighted gradient echo sequence (TR = 2,000 ms, TE = 35 ms, image matrix = 64 × 64, FOV = 224 mm, flip angle = 80°, slice thickness = 3.0 mm, distance factor = 17%, voxel size = 3.5 × 3.5 × 3.5 mm^3^, 30 axial slices). In the scanner, stimuli were projected onto a screen at the end of the magnet bore and participants viewed the stimuli through an angled mirror located above their eyes on the head coil. Stimulus presentation was controlled by E-Prime 2.0 (www.pstnet.com/eprime; Psychology Software Tools) running under Frames XP. Participants were placed head first and supine in the scanner bore. They were instructed not to move their heads to avoid motion artifacts and foam cushions were placed to minimize head movements.

### Image processing

The fMRI data were preprocessed and analyzed using SPM12 (Wellcome Department of Cognitive Neurology, London, UK). Before statistical analysis, data were preprocessed to remove sources of noise and artifact. Inhomogeneities in the magnetic field were corrected using the fieldmap (Cusack & Papadakis, 2002), slice-time correction was applied to amend differences in acquisition time between slices for each whole-brain volume, and realigned within and across runs for the removal of the movement effects. The functional data were then transformed into a standard anatomical space (2-mm isotropic voxels) based on the ICBM152 brain template (Montreal Neurological Institute). Normalized data were then spatially smoothed (6-mm full-width at half-maximum, FWHM) using a Gaussian Kernel. Finally, because there is no widely accepted method to isolate and remove movement artifacts from the neural signal in event-related designs (Caballero-Gaudes & Reynolds, 2017; Ciric et al., 2017; Satterthwaite et al., 2013) and because studies with nearly motionless participants (i.e., healthy undergraduates) may not benefit much from more complex procedures (Satterthwaite et al., 2013), we adopted a conservative approach by applying the standard SPM12 procedure in which six movement regressors are included to the design and an additional procedure in which noisy motion signals are detected and removed from the time series using “spike regression.” This removal procedure provides a single regressor for each outlier motion spike and appears to be quite effective (Ciric et al., 2017; Power et al., 2014; Satterthwaite et al., 2013). In particular, the preprocessed data were examined, using the Artifact Detection Tool software package (ART; http://web.mit.edu/swg/art/art.pdf;http://www.nitrc.org/projects/artifact_detect/), for excessive motion artifacts and for correlations between motion and experimental design, and between global mean signal and experimental design. Outliers were identified in the temporal differences series by assessing between-scan differences (Z-threshold: 3.0 mm, scan to scan movement threshold: 0.5 mm; rotation threshold: 0.02 radians). These outliers were omitted in the analysis by including a single regressor for each outlier. No systematic correlations between motion and experimental design or global signal and experimental design were identified. Six directions of motion parameters from the realignment step as well as outlier time points (defined by ART) were included as nuisance regressors in the design. We used a default high-pass filter of 128s and serial correlations were accounted for by the default auto-regressive AR (1) model.

### Statistical analyses

To test our specific predictions, both in the behavioral and fMRI data analysis we analyze the belief conditions separately for (a) the Story phase, (b) the Question phase for the self, and (c) the Question phase for the smurf. Specifically, we compared all belief conditions against each other (Similar True, Mixed True, Mixed False, Similar False). Note, however, that during the story phase or when taking a self-perspective, the Mixed True and Mixed False conditions are collapsed, because they designate the truth only from the smurf’s perspective and hence smurfs play an identical role here.

#### Behavioral data

We analyzed the error rates and response times (RT; correct trials only and after excluding outliers beyond two and a half standard deviations from the mean of each condition) from the question phase. To test our specific hypotheses, for each of the perspectives (Self and Smurf), differences between conditions were tested using an analysis of variance (ANOVA), with Condition as within-participant factor (Similar True, Mixed True, Mixed False, Similar False; or with the Mixed conditions combined in the Story and Self phases). Significant ANOVAs were further explored using paired samples t-tests with *p* < 0.05 (two-tailed).

#### Imagining data

For the first level (individual) analysis, the onset regressors were defined and analyzed for each condition (Similar True, Mixed True, Mixed False, Similar False) separately for the Story and Question phase (Self and Smurf). During the story phase, participants would understand that the smurfs could potentially develop a false belief as soon as they would leave the screen. At the question phase, participants would have to select the appropriate belief of the smurfs or themselves. Therefore, the belief formation onset was defined at the beginning of the videos (when the smurfs started to move away) and the question onset right before the appearance of the question bar. We reasoned that it would take some time to follow the story and understand its mentalizing implications, whereas when a question appears after the story, mentalizing inferences should be readily available. Therefore, during the story phase, event duration after onset was set to 4 seconds (roughly the duration of the whole story) and during the question phase duration was set to 0 seconds. To verify this, we estimated activity given 0- and 4-second durations in both phases, and the analysis confirmed that these *a priori* specifications for duration yielded the strongest effects. A canonical hemodynamic response function was used to model the hemodynamic response to each type of event. The six head movement parameters also were included in the model as nuisance vectors. Each condition of interest was estimated for each participant, and extracted for the second level analysis.

In the second level (group) analysis, we computed different contrast of interests for different perspectives and beliefs as noted above. That is, we analyzed the Story and Question phase (for Self and Smurf) separately and computed contrasts between all conditions (Similar True, Mixed True, Mixed False, Similar False). Although for the Story and Self conditions, the Mixed conditions were collapsed, because they were undistinguishable for the participants (and contrast weights were appropriately adjusted so that the weights summed to 0). An initial cluster threshold of *p* < 0.001 (voxel-wise, uncorrected) was used with a minimum cluster extent of 10 voxels to define the clusters, and significance was tested with a cluster FWE-corrected threshold of *p* < 0.001. In addition, percent signal changes were computed for *a priori* regions of interest (ROI) using MarsBar. These ROIs were centered around on coordinates from the meta-analyses by Van Overwalle (2009) and Van Overwalle and Baetens (2009; see also Ma et al., 2012), using a sphere with a radius of 8 mm, and included the bilateral TPJ (MNI coordinates: ±50 -50 25), pmFC (0 20 45) and bilateral IFG (±40 25 20). We also used similar *a priori* coordinates of the TPJ from the 15 false belief studies in the meta-analysis by Schurz et al. (2014; -55 -65 27; 56 -56 25) and the mPFC coordinates from van der Meer et al. (2011; -6 16 46) and *a posteriori* from this study (-4 14 56). We selected the coordinates with the strongest differences between belief conditions, and this included all coordinates from the Van Overwalle lab, except the TPJ coordinates from Schurz et al. (2014), which showed stronger effects during the story phase and were used instead. Differences between conditions were tested using an ANOVA, and contrasts between conditions were further explored using simple *t*-tests with *p* < 0.05.

## Results

We analyzed the belief conditions separately for the Story phase and the Question phase (Smurf and Self). Our prediction was that when taking a smurf’s perspective, processing and activation would increase given more false beliefs held by the smurfs. This should show up in longer behavioral responses and higher fMRI activation. Note that during the Story and self-perspective Question phase, the Mixed True and Mixed False beliefs are collapsed, because the two conditions are indistinguishable for the participants as the two smurfs play a similar role, because the target of judgment is only indicated afterwards. (Statistics of all *t*-tests including *t*-values and *p*-values, are listed in Supplementary Table 2).

### Behavioral results

**Fig. 2.**
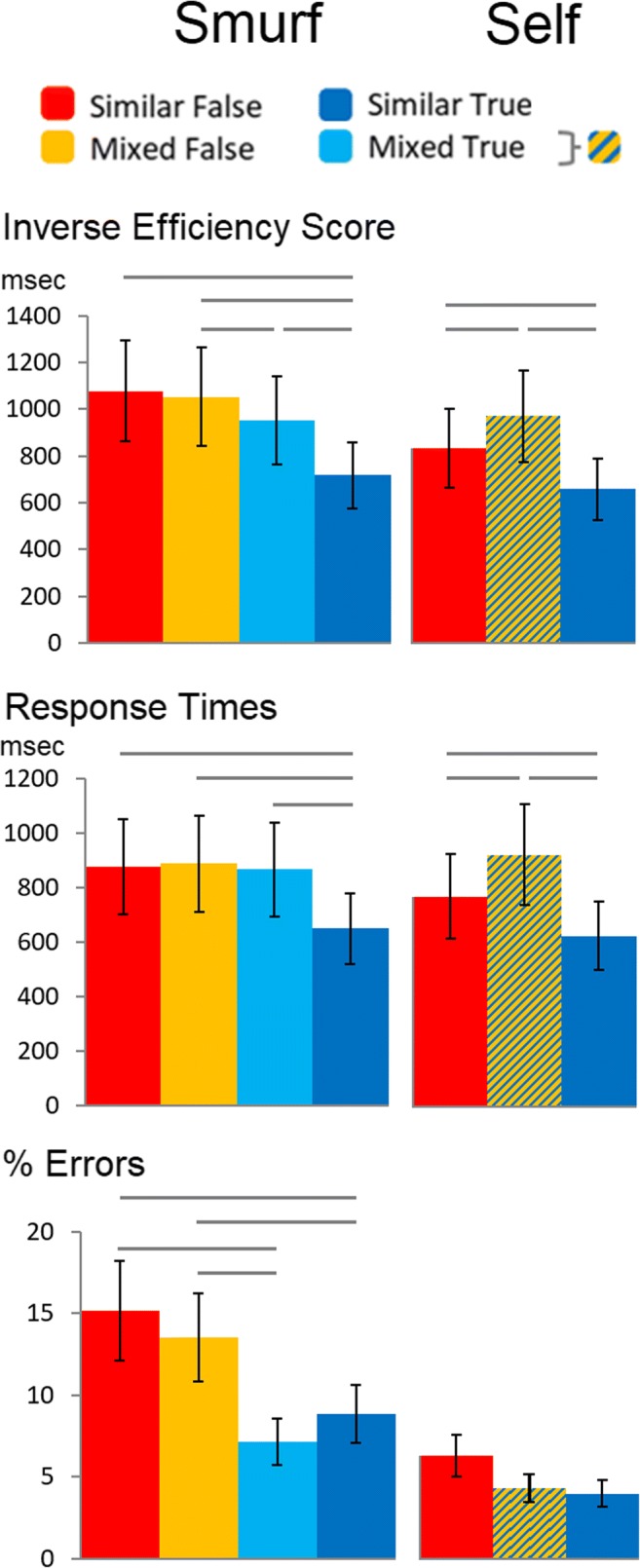
Mean inverse efficiency score, response times and error rates in function of condition during the Question phase. Horizontal lines denote conditions that differ with *p* ≤ 0.05 using paired *t*-tests

Participants’ RTs and error rates for correct responses during the Question phase were combined in an Inverse Efficiency Score (IES) per condition (Similar True, Mixed True, Mixed False, Similar False; with the Mixed conditions combined in the Self question phase). An IES (Bruyer & Brysbaert, 2011) adjusts the response times by dividing them by the accuracy ratio, so that less correct responses are penalized by slower efficiency scores (Fig. 2).

With respect to the smurf’s perspective, an ANOVA on IESs revealed a main effect of Belief condition, *F*(3, 72) = 20.5, *p* < 0.001, with means showing an increase in IESs given more agents holding false beliefs. Simple t-tests showed that IESs were significantly different between all conditions except the Mixed False and Similar False conditions, with the Similar True condition faster than the Mixed True condition, *t*(24) = 5.61, *p* < 0.001, which was faster than the Mixed False condition, *t*(24) = 2.47, *p* < 0.05. With respect to a self-perspective, the ANOVA on IESs revealed a very strong main effect, *F*(2, 48) = 53,18, *p* < 0.001. Simple t-tests showed an unexpected pattern in that IESs were significantly slower after Mixed as opposed to Similar False or True beliefs, *t*(24) = 4.24-8.62, *p* < 0.001. Similar True beliefs were the fastest overall in comparison with the other conditions, *t*(24) = 8.39-8.62, *p* < 0.001.

The pattern of results shows that when taking a smurf’s perspective, there was a linear effect of number of agents holding false beliefs as we predicted, except that there were no differences between the Similar False and Mixed False conditions. This pattern was absent when taking a self-perspective. Note that the pattern of results for the raw response times was very similar, although the increasing effect given a smurf’s perspective was less evident.

### fMRI results

**Fig. 3.**
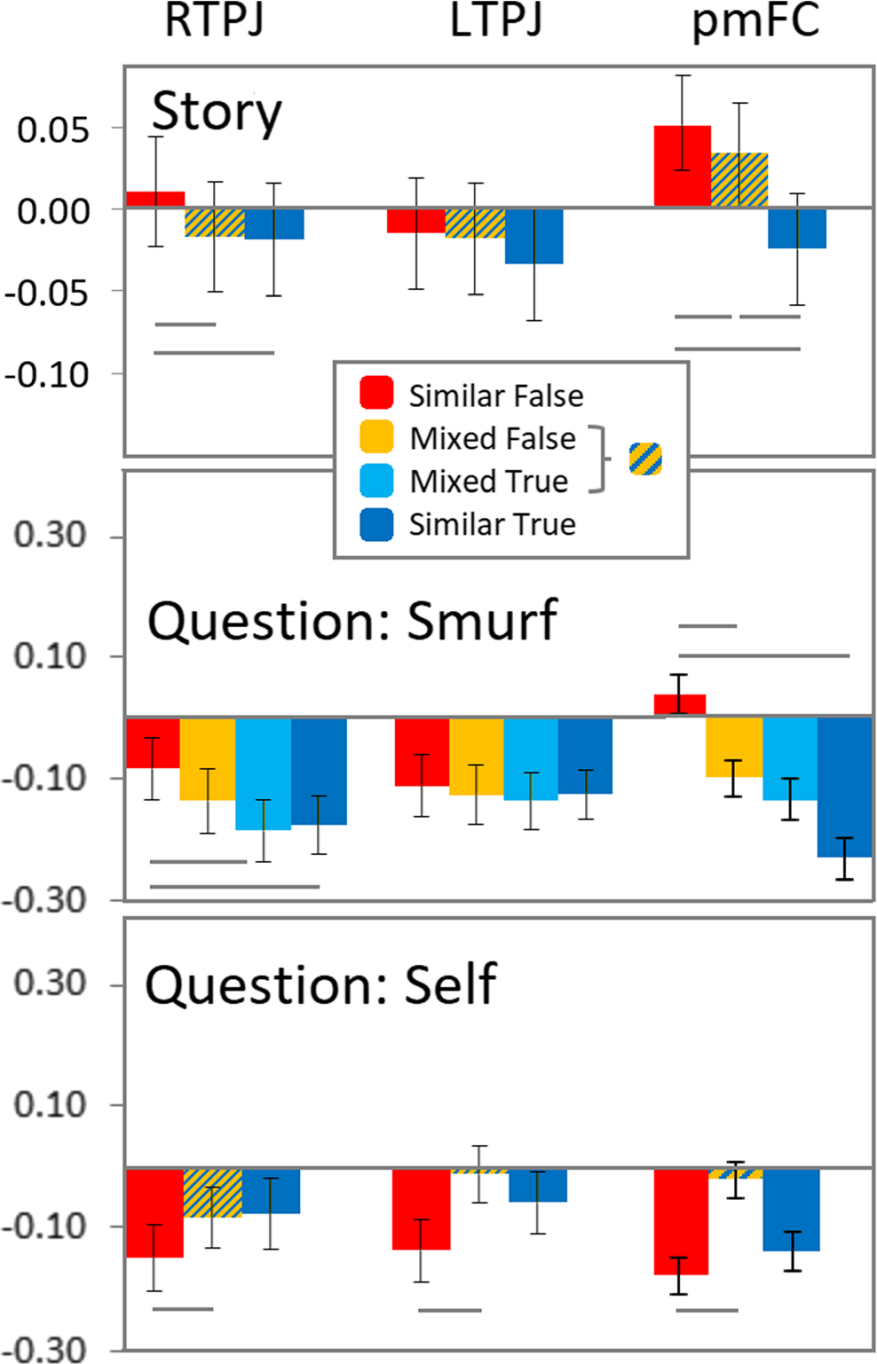
Percentage signal change during story and question (Smurf and Self) phase for mentalizing and conflict monitoring areas. Horizontal lines denote conditions that differ with *p* ≤ 0.05 using paired *t*-tests. TPJ = temporo-parietal junction (L = left, R = right; MNI coordinates: -55 -65 27; 56 -56 25), pmFC = posterior frontal cortex (MNI 0 20 45)

We computed whole-brain contrasts for each phase and perspective. In addition, to test our prediction of a linearly increasing pattern of activation, we analyzed the percentage signal change of *a priori* ROIs in the bilateral TPJ and pmFC (Fig. 3).

#### Whole brain analysis

The whole brain analysis of the Story Phase revealed a significant activation of the pmFC in the comparison of Similar False Beliefs > Similar True Beliefs, as well as Mixed True & False Beliefs > Similar True Beliefs (Supplementary Table 1, Supplementary Figure 1). Other areas revealed also significant activation in one of these comparisons: The Mixed True & False Belief > Similar True Belief showed activation in the bilateral IFG, bilateral precuneus, bilateral superior parietal cortex, left insula, right caudate, bilateral putamen, and left middle occipital cortex. Unexpectedly, in the opposite direction, the Mixed True & False Belief > Similar False Belief contrast showed activation in the left superior frontal gyrus, and the Similar True Belief > Similar False Belief contrast revealed activation in the medial cuneus and medial calcarine gyrus. The analysis of the Question phase revealed no activation above threshold, even with a more lenient FWE-corrected cluster threshold of *p* < 0.05.

#### ROI analysis

In the Story Phase, an ANOVA on the percentage signal change revealed a main effect in the right TPJ, *F*(2, 48) = 4.26, *p* < 0.05, and pmFC, *F*(2, 48) = 12.79, *p* < 0.001, with means showing an increase in activation going from Similar True, Mixed True & False to Similar False. Simple t-tests confirmed that in the right TPJ, the Similar False condition was significantly stronger than the other two conditions, *p* < 0.05, which did not differ from each other. In the pmFC, all conditions differed from each other, *p* < 0.05. There was no significant main effect in the left TPJ (Fig. 3). For exploratory reasons, we tested also the bilateral IFG and found that Similar False Beliefs were most active, sometimes significantly more so than some other Belief conditions, *p* < 0.05 (Supplementary Figure 2).

For the smurf’s perspective in the Question phase, an ANOVA on the percentage signal change revealed a main effect in the right TPJ, *F*(3, 72) = 2.70, *p* = 0.052, and pmFC, *F*(3, 72) = 4.32, *p* < 0.01, with means showing an increase in activation going from Similar True, Mixed True, Mixed False to Similar False. Simple t-tests largely confirmed this pattern, although not all conditions differed significantly between each other. In the right TPJ, the Similar False condition was significantly stronger than the Mixed True and Similar True conditions only, *p* < 0.05. In the mPFC, the Similar False condition was significantly stronger than the Mixed False and Similar True conditions, *p* < 0.05, and marginally so for the Mixed True condition, *p* = 0.055. There was no significant main effect in the left TPJ (Fig. 3). The bilateral IFG showed again that Similar False Beliefs were most active, sometimes significantly more so than other Belief conditions (Supplementary Figure 2).

For a self-perspective, the signal change analysis revealed a significant main effect in the pmFC, *F*(2, 48) = 3.44, *p* < 0.05. However, simple *t*-tests revealed that the activation in the Similar False condition was significantly *lower* than in the Mixed True & False condition in the bilateral TPJ and pmFC, while the latter condition did not differ significantly from the Similar True condition (Fig. 3). These data seem to suggest that the TPJ and pmFC are more activated given a self-perspective when at least one smurf has a belief similar to the self. The bilateral IFG showed similar effects (Supplementary Figure 2).

For exploratory reasons, we also analyzed the dorsal and ventral part of the medial prefrontal cortex which are key areas in mentalizing (Schurz et al., 2014; Van Overwalle, 2009), but found little modulation in any of the perspectives or phases (Supplementary Figure 2).

## Discussion

An important limitation of past research on mentalizing is that false beliefs were investigated almost exclusively for a single agent, either alone (Bardi et al., 2017; Özdem, Brass, et al., 2017; Schuwerk et al., 2014) or in the context of another agent who always held true beliefs (Saxe & Kanwisher, 2003). To investigate the role of number of false beliefs in a more systemically manner, this study investigated for the first time distinct beliefs held by two agents by varying all combinations of their true and false beliefs. Moreover, to enforce the computation of beliefs to each agent, we distinguished between an initial Story phase, in which all the perspectives of all agents (smurfs and self) had to be considered, and a subsequent Question phase, in which a single perspective had to be taken (see also Schuwerk et al., 2014). Our key prediction was that false beliefs held by more agents would generate increasingly more TPJ activation when taking the agent’s perspective. In addition, given that one’s self-perspective was prominent, we also expected an increase of “decoupling” or inhibition of one’s own perspective on the truth in favor of the false beliefs held by the agents, eliciting a similar increasing pattern of executive control in the pmFC.

The behavioral results revealed an increase in response times (adjusted for accuracy) given false beliefs held by more agents when taking an agent’s perspective, resulting in the slowest responses in the mixed or shared false belief conditions which did not differ. These results give some support for our hypothesis. This pattern of results was completely absent when taking a self-perspective and rather showed the slowest response time in the mixed condition.

### TPJ and switching to internal thoughts

The increasing pattern of activation in the right TPJ given false beliefs held by more agents supports our prediction and the crucial role of the TPJ in orienting to the belief of each agent. Of critical importance is that during the Story phase, two agents holding false beliefs revealed stronger right TPJ activation than if one of these agents held a false belief, or none of the agents held false beliefs. That is, we found an additional effect of a second false belief on right TPJ activity over and above the effect of just one false belief. Our results are consistent with Schuwerk et al. (2014) who used a very similar paradigm with a single agent, as well as much prior work on false belief reasoning (see meta-analyses by Schurz et al., 2014; Van Overwalle, 2009; Van Overwalle & Baetens, 2009). The increasing pattern of TPJ activation when false beliefs are held by more agents confirms the role of the TPJ in reorienting attention to other minds, especially when there is more than one mind to consider (Cabeza et al., 2012). As stated by Bardi et al. (2017), the TPJ is associated “with self-other distinction, i.e., the ability to keep self and other representations apart and switch between them.” Apparently, our pattern of increasing activation demonstrates that this reorientation process is graded and increasingly more applied when more agents hold false beliefs, probably because this entails a greater reliance on and switching to our inner thoughts about false beliefs in comparison with a focus on reality (Cabeza et al., 2012).

However, a limitation of the present paradigm, as well as prior research, is that most often only two belief states are possible (e.g., left or right frame as in our study; or two story responses as in prior research). To provide a correct response on a false belief question, it is sufficient to switch one’s perspective away from the truth response option towards the alternate response option (Cabeza et al., 2012), without considering the full content of the false belief. Even in the present work, when one or two agents hold similar or opposing beliefs, a correct response can simply be obtained by tagging each agent’s presence or belief as true or false and switching from the truth to the alternative response option when the belief is false. This can be accomplished without processing the belief content itself. Further research is needed to investigate whether switching between perspectives and simply tagging presence/beliefs as true or false versus elaborating on the content of these beliefs and holding these in one’s mind are related or distinct neural processes recruiting the same or distinct brain areas.

### pmFC and decoupling from one’s self-perspective on reality

As interesting aspect of the present paradigm is that an increase in activation of the pmFC was observed given false beliefs held by more agents, in a similar increasing pattern as the TPJ. In most false belief research, activation of the mPFC is not revealed, unless one’s self-perspective is made more salient (Hartwright et al., 2015; van der Meer et al., 2011). This suggests that the pmFC reflects the discrepancy between own and other perspective or beliefs. Indeed, false beliefs require participants inhibiting their own knowledge about the true state of reality to understand the false beliefs by other agents. In the present study, one’s self-perspective was salient, because the participants had to consider both their own and the agent’s perspectives when the animation unfolded and were thus forced to keep discrepant true and false beliefs in mind, but strictly apart. The fact that an agent’s perspective was requested only after the animation ended may have further increased this discrepancy and the role the pmFC played in our study.

The increasing pattern of activation in the pmFC given more discrepant false beliefs is consistent with the view that false belief reasoning involves a process of “decoupling” whereby participants inhibit their self-beliefs in order to understand the discrepant mental beliefs of others. We argue that decoupling is a form of conflict processing and resolution (Botvinick et al., 2004; Shenhav, Cohen, & Botvinick, 2016), in which the pmFC is associated with conflict monitoring (i.e., observing a discrepancy between the beliefs of self and other), thereby allocating optimal executive control in the lateral IFG to resolve the conflict. One obvious way of conflict resolution is by attributing or “tagging” false beliefs to individual agents and preserve that information in working memory until a response on a specific perspective can be provided.

This interpretation is plausible given recent evidence from Meyer, Spunt, Berkman, Taylor, and Lieberman (2012); Meyer, Taylor, and Lieberman (2015), indicating that holding social information in working memory activates executive brain areas including the pmFC and bilateral IFG, and that this activation grows stronger with an increasing amount of social information that needs to be memorized. Their study required participants to mentally rank two, three, or four persons according to a given trait (e.g., funny). The results showed that ranking an increasing number of persons on a social dimension augmented activation in the executive control network encompassing the pmFC and IFG. It should be noted, however, that Samson and colleagous (Samson, Houthuys, & Humphreys, 2015) challenged the idea that self-perspective inhibition is a domain-general executive process; they demonstrated that deficits in self-inhibition are dissociated from deficits in classic executive tasks. Moreover, we note that while the pmFC showed a clear increasing pattern of activation when dealing with more agents holding false beliefs, this pattern was reduced in the bilateral IFG showing only increased activation for false beliefs held by two agents. More research is needed to investigate the distinct roles of the pmFC and the IFG in social memory processing.

### Taking a self-perspective

When taking a self-perspective, we observed a very unexpected pattern of results. We found increasing activation when the agents held mixed beliefs and the least activation when both agents held false beliefs. This pattern suggests that situations in which agents hold mixed beliefs appear most conflicting for the self in contrast to less ambiguous situations where agents hold beliefs that are entirely (dis)similar to the self. This suggests an unexpected impact of other perspectives on reality processing by the self, which also was revealed in previous research on automatic perspective taking (Qureshi, Apperly, & Samson, 2010; Ramsey, Hansen, Apperly, & Samson, 2013). Because this is the first time that a self-perspective was analyzed in a false belief task, more research is needed to investigate the robustness of the present results.

## Conclusions

We developed a novel false belief task by having two agents instead of a single agent holding false or true beliefs, with the goal to better understand the mechanisms underlying false belief mentalizing. We found that the TPJ is increasingly activated when participants track false beliefs of two agents rather than a single agent or no one. However, this pattern of results only occurs when participants take the perspective of the agent (as in classic false belief tasks). This increasing activation of the TPJ suggest increasing processing costs to reorient to false beliefs of multiple agents. We also found a similar pattern in the pmFC, suggesting that false beliefs by multiple agents are perceived as increasingly conflicting with one’s self-perspective on reality (Botvinick et al., 2004; Shenhav et al., 2016). When taking a self-perspective, we found most activation when the other agents held mixed beliefs, suggesting that this situation is perhaps most ambiguous with respect to the self.

## Electronic supplementary material

ESM 1 (DOCX 372 kb)
