# Supplementary material for: The neural representation of mental beliefs held by two agents
Source: Cogn Affect Behav Neurosci. 2019 Apr 12;19(6):1433–43. doi: 10.3758/s13415-019-00714-2 (PMC6861364; doi:10.3758/s13415-019-00714-2)
Supplement: Supplementary file 1 — (DOCX 372 kb) [file 13415_2019_714_MOESM1_ESM.docx]

| Supplementary Table 1: Whole-brain analysis (correct trials only) | | | | | | | |
| --- | --- | --- | --- | --- | --- | --- | --- |
| Phase, Comparison and Anatomical Label | | | voxels | x | y | z | t |
|  |  |  |  |  |  |  |  |
| **Story Phase: Comparisons between Belief Conditions (4 sec)** | | | | |  |  |  |
|  | ***Similar False Belief > Similar True Belief*** | |  |  |  |  |  |
|  |  | L Posterior Medial Frontal (pmFC) | 144 | -6 | 8 | 58 | 4.099 |
|  |  | L Posterior Medial Frontal (pmFC) |  | -6 | 8 | 68 | 3.570 |
|  |  | L Posterior Medial Frontal (pmFC) |  | -10 | 10 | 46 | 3.265 |
|  |  |  |  |  |  |  |  |
|  | ***Mixed True & False Belief > Similar True Belief*** | | |  |  |  |  |
|  |  | L Middle Frontal Gyrus (L IFG) | 768 | -26 | -4 | 52 | 5.806 |
|  |  | L Middle Frontal Gyrus (L IFG) |  | -28 | -6 | 40 | 3.888 |
|  |  | L Precentral Gyrus (L IFG) |  | -50 | 0 | 40 | 3.875 |
|  |  | L Posterior Medial Frontal (pmFC) | 567 | -4 | 8 | 56 | 5.726 |
|  |  | L Posterior Medial Frontal (pmFC) |  | -4 | 2 | 72 | 3.768 |
|  |  | R Superior Frontal Gyrus (R IFG) | 432 | 24 | -2 | 52 | 4.800 |
|  |  | R Precentral Gyrus (R IFG) |  | 32 | -6 | 54 | 4.256 |
|  |  | R Superior Frontal Gyrus (R IFG) |  | 32 | 2 | 64 | 3.632 |
|  |  | L Precuneus | 470 | -12 | -62 | 62 | 4.726 |
|  |  | L Superior Parietal Lobule |  | -24 | -60 | 58 | 3.874 |
|  |  | L Superior Parietal Lobule |  | -14 | -64 | 48 | 3.853 |
|  |  | L Insula | 186 | -30 | 24 | -8 | 4.706 |
|  |  | L Insula |  | -24 | 18 | -8 | 4.113 |
|  |  | L Insula |  | -30 | 18 | 6 | 3.301 |
|  |  | R Precuneus | 238 | 12 | -56 | 52 | 4.565 |
|  |  | R Superior Parietal Lobule |  | 22 | -60 | 54 | 3.745 |
|  |  | R Precuneus |  | 12 | -68 | 54 | 3.608 |
|  |  | R Caudate Nucleus | 259 | 12 | 4 | 0 | 4.484 |
|  |  | R Caudate Nucleus |  | 8 | 12 | 2 | 3.793 |
|  |  | R Putamen |  | 18 | 18 | -6 | 3.433 |
|  |  | L Pallidum | 136 | -16 | 6 | 2 | 3.972 |
|  |  | L Putamen |  | -16 | 14 | 0 | 3.319 |
|  |  | L Middle Occipital Gyrus | 132 | -26 | -66 | 30 | 3.926 |
|  |  | L Middle Occipital Gyrus |  | -28 | -76 | 34 | 3.672 |
|  |  |  |  |  |  |  |  |
|  | ***Mixed True & False Belief > Similar False Belief*** | | |  |  |  |  |
|  |  | L Superior Frontal Gyrus | 124 | -24 | -4 | 56 | 4.106 |
|  |  |  |  |  |  |  |  |
|  | ***Similar True Belief > Similar False Belief*** | |  |  |  |  |  |
|  |  | L Cuneus | 449 | -2 | -84 | 18 | 4.322 |
|  |  | R Cuneus |  | 12 | -78 | 24 | 4.129 |
|  |  | R Calcarine Gyrus |  | 4 | -78 | 16 | 3.957 |
|  |  |  |  |  |  |  |  |
| **Question Phase: Comparisons between Belief Conditions for the Self or Smurf (0 sec)** | | | | | | | |
|  |  |  |  |  | --- |  |  |
|  |  |  |  |  |  |  |  |
| **Question Phase: Comparisons between Perspectives (Smurf > Self; 0 sec)** | | | | | | |  |
|  |  |  |  |  | --- |  |  |
|  |  |  |  |  |  |  |  |
| *Note*: x, y, and z = Montreal Neurological Institute (MNI) coordinates of the peak values; t = t-score of the peak values; R = Right, L = Left, pmFC = posterior medial frontal cortex; IFG = inferior frontal gyrus. Whole brain analysis with *p* < .001 (uncorrected) and cluster extent > 10 voxels. Listed are clusters with FWE-corrected *p* < .001. All whole-brain contrasts not shown are below threshold. The Mixed conditions are collapsed for the Story phase and Self Question phase. | | | | | | | |

**Supplementary Table 2**: Statistical values of the t-tests between belief conditions for the behavioral and fMRI – ROI analyses.

|  |  |  |  |  |  |  |  |  |
| --- | --- | --- | --- | --- | --- | --- | --- | --- |
| **BEHAVIORAL DATA: SELF PERSPECTIVE** | | | | | | | | |
|  |  |  |  |  |  |  |  |  |
|  |  | **p-value** |  |  |  | **t-value(df=25-1)** | |  |
|  |  |  |  |  |  |  |  |  |
| **IES** |  | **2FBs_Self** | **Mixed_Self** |  |  | **2FBs_Self** | **Mixed_Self** |  |
|  | 2TBs_Self | 0.000 | 0.000 |  |  | -8.393 | -8.624 |  |
|  | Mixed_Self | 0.000 |  |  |  | -4.291 |  |  |
|  |  |  |  |  |  |  |  |  |
| **Response Time** | |  |  |  |  |  |  |  |
|  | 2TBs_Self | 0.000 | 0.000 |  |  | -7.635 | -11.136 |  |
|  | Mixed_Self | 0.000 |  |  |  | -8.170 |  |  |
|  |  |  |  |  |  |  |  |  |
| **Error Rate** | |  |  |  |  |  |  |  |
|  | 2TBs_Self | 0.060 | 0.836 |  |  | -1.977 | -0.209 |  |
|  | Mixed_Self | 0.270 |  |  |  | -1.130 |  |  |
|  |  |  |  |  |  |  |  |  |
| **BEHAVIORAL DATA: SMURF PERSPECTIVE** | | | | | | | | |
|  |  |  |  |  |  |  |  |  |
|  |  | **p-value** |  |  |  | **t-value(df=25-1)** | |  |
| **IES** |  | **2FBs_Smurf** | **Mixed_FB_Smurf** | **Mixed_TB_Smurf** |  | **2FBs_Smurf** | **Mixed_FB_Smurf** | **Mixed_TB_Smurf** |
|  | 2TBs_Smurf | 0.000 | 0.000 | 0.000 |  | -5.824 | -7.087 | -5.608 |
|  | Mixed_TB_Smurf | 0.069 | 0.021 |  |  | -1.907 | -2.468 |  |
|  | Mixed_FB_Smurf | 0.580 |  |  |  | -0.561 |  |  |
|  |  |  |  |  |  |  |  |  |
| **Response Time** | |  |  |  |  |  |  |  |
|  | 2TBs_Smurf | 0.000 | 0.000 | 0.000 |  | -8.878 | -8.058 | -9.824 |
|  | Mixed_TB_Smurf | 0.683 | 0.334 |  |  | -0.414 | -0.987 |  |
|  | Mixed_FB_Smurf | 0.612 |  |  |  | -0.514 |  |  |
|  |  |  |  |  |  |  |  |  |
| **Error Rate** | |  |  |  |  |  |  |  |
|  | 2TBs_Smurf | 0.006 | 0.007 | 0.332 |  | -3.038 | -2.947 | -0.990 |
|  | Mixed_TB_Smurf | 0.001 | 0.001 |  |  | -3.674 | -3.647 |  |
|  | Mixed_FB_Smurf | 0.284 |  |  |  | -1.095 |  |  |
|  |  |  |  |  |  |  |  |  |
| **FMRI DATA** | | | | | | | | |
|  |  |  |  |  |  |  |  |  |
| **STORY PHASE** | | | | | | | | |
|  |  |  |  |  |  |  |  |  |
|  |  | **p-values** |  |  |  | **t-values** |  |  |
| **RTPJ (Schurz)** | | **Mixed** | **2TBs** |  |  | **Mixed** | **2TBs** |  |
|  | **2FBs** | 0.008 | 0.033 |  |  | 2.895 | 2.257 |  |
|  | **Mixed** |  | 0.880 |  |  |  | 0.153 |  |
|  |  |  |  |  |  |  |  |  |
| **LTPJ (Schurz)** | | **Mixed** | **2TBs** |  |  | **Mixed** | **2TBs** |  |
|  | **2FBs** | 0.770 | 0.231 |  |  | 0.295 | 1.230 |  |
|  | **Mixed** |  | 0.268 |  |  |  | 1.134 |  |
|  |  |  |  |  |  |  |  |  |
| **vmPFC** |  | **Mixed** | **2TBs** |  |  | **Mixed** | **2TBs** |  |
|  | **2FBs** | 0.283 | 0.026 |  |  | 1.098 | 2.376 |  |
|  | **Mixed** |  | 0.057 |  |  |  | 2.000 |  |
|  |  |  |  |  |  |  |  |  |
| **dmPFC** |  | **Mixed** | **2TBs** |  |  | **Mixed** | **2TBs** |  |
|  | **2FBs** | 0.259 | 0.168 |  |  | 1.156 | 1.423 |  |
|  | **Mixed** |  | 0.029 |  |  |  | 2.325 |  |
|  |  |  |  |  |  |  |  |  |
| **pmFC** |  | **Mixed** | **2TBs** |  |  | **Mixed** | **2TBs** |  |
|  | **2FBs** | 0.050 | 0.000 |  |  | 2.067 | 4.837 |  |
|  | **Mixed** |  | 0.009 |  |  |  | 2.850 |  |
|  |  |  |  |  |  |  |  |  |
| **RIFG** |  | **Mixed** | **2TBs** |  |  | **Mixed** | **2TBs** |  |
|  | **2FBs** | 0.115 | 0.168 |  |  | 1.634 | 1.423 |  |
|  | **Mixed** |  | 0.857 |  |  |  | 0.182 |  |
|  |  |  |  |  |  |  |  |  |
| **LIFG** |  | **Mixed** | **2TBs** |  |  | **Mixed** | **2TBs** |  |
|  | **2FBs** | 0.100 | 0.004 |  |  | 1.709 | 3.209 |  |
|  | **Mixed** |  | 0.012 |  |  |  | 2.730 |  |
|  |  |  |  |  |  |  |  |  |
| **QUESTION PHASE: SMURF PERSPECTIVE** | | | | | | | | |
|  |  |  |  |  |  |  |  |  |
|  |  | **p-values** |  |  |  | **t-values** |  |  |
| **RTPJ (Schurz)** | | **MixFB_Smurf** | **MixTB_Smurf** | **2TBs_Smurf** |  | **MixFB_Smurf** | **MixTB_Smurf** | **2TBs_Smurf** |
|  | **2FBs_Smurf** | 0.241 | 0.011 | 0.030 |  | 1.203 | 2.747 | 2.305 |
|  | **MixFB_Smurf** |  | 0.130 | 0.401 |  |  | 1.567 | 0.855 |
|  | **MixTB_Smurf** |  |  | 0.811 |  |  |  | 0.242 |
|  |  |  |  |  |  |  |  |  |
| **LTPJ (Schurz)** | |  |  |  |  |  |  |  |
|  | **2FBs_Smurf** | 0.815 | 0.536 | 0.808 |  | 0.236 | 0.628 | 0.246 |
|  | **MixFB_Smurf** |  | 0.875 | 0.989 |  |  | 0.158 | 0.014 |
|  | **MixTB_Smurf** |  |  | 0.821 |  |  |  | 0.229 |
|  |  |  |  |  |  |  |  |  |
| **vmPFC** |  | **MixFB_Smurf** | **MixTB_Smurf** | **2TBs_Smurf** | |  |  |  |
|  | **2FBs_Smurf** | 0.979 | 0.589 | 0.401 |  | 0.027 | 0.548 | 0.855 |
|  | **MixFB_Smurf** |  | 0.604 | 0.280 |  |  | 0.525 | 1.104 |
|  | **MixTB_Smurf** |  |  | 0.141 |  |  |  | 1.522 |
|  |  |  |  |  |  |  |  |  |
| **dmPFC** |  | **MixFB_Smurf** | **MixTB_Smurf** | **2TBs_Smurf** | |  |  |  |
|  | **2FBs_Smurf** | 0.543 | 0.089 | 0.436 |  | 0.617 | 1.772 | 0.792 |
|  | **MixFB_Smurf** |  | 0.245 | 0.266 |  |  | 1.193 | 1.140 |
|  | **MixTB_Smurf** |  |  | 0.023 |  |  |  | 2.435 |
|  |  |  |  |  |  |  |  |  |
| **pmFC** |  | **MixFB_Smurf** | **MixTB_Smurf** | **2TBs_Smurf** | |  |  |  |
|  | **2FBs_Smurf** | 0.021 | 0.055 | 0.001 |  | 2.474 | 2.020 | 3.749 |
|  | **MixFB_Smurf** |  | 0.685 | 0.103 |  |  | 0.411 | 1.697 |
|  | **MixTB_Smurf** |  |  | 0.218 |  |  |  | 1.264 |
|  |  |  |  |  |  |  |  |  |
| **RIFG** |  | **MixFB_Smurf** | **MixTB_Smurf** | **2TBs_Smurf** | |  |  |  |
|  | **2FBs_Smurf** | 0.005 | 0.003 | 0.038 |  | 3.104 | 3.288 | 2.191 |
|  | **MixFB_Smurf** |  | 0.357 | 0.271 |  |  | 0.940 | 1.128 |
|  | **MixTB_Smurf** |  |  | 0.101 |  |  |  | 1.703 |
|  |  |  |  |  |  |  |  |  |
| **LIFG** |  | **MixFB_Smurf** | **MixTB_Smurf** | **2TBs_Smurf** | |  |  |  |
|  | **2FBs_Smurf** | 0.009 | 0.100 | 0.002 |  | 2.848 | 1.712 | 3.388 |
|  | **MixFB_Smurf** |  | 0.510 | 0.969 |  |  | 0.669 | 0.039 |
|  | **MixTB_Smurf** |  |  | 0.620 |  |  |  | 0.502 |
|  |  |  |  |  |  |  |  |  |
| **QUESTION PHASE: SELF PERSPECTIVE** | | | | | | | | |
|  |  |  |  |  |  |  |  |  |
|  |  | **p-values** |  |  |  | **t-values** |  |  |
| **RTPJ (Schurz)** | | **Mixed_Self** | **2TBs_Self** |  |  | **Mixed_Self** | **2TBs_Self** |  |
|  | **2FBs_Self** | 0.024 | 0.171 |  |  | 2.411 | 1.412 |  |
|  | **Mixed_Self** |  | 0.869 |  |  |  | 0.167 |  |
|  |  |  |  |  |  |  |  |  |
| **LTPJ (Schurz)** | |  |  |  |  |  |  |  |
|  | **2FBs_Self** | 0.012 | 0.203 |  |  | 2.720 | 1.308 |  |
|  | **Mixed_Self** |  | 0.469 |  |  |  | 0.736 |  |
|  |  |  |  |  |  |  |  |  |
| **vmPFC** |  | **Mixed_Self** | **2TBs_Self** |  |  |  |  |  |
|  | **2FBs_Self** | 0.843 | 0.189 |  |  | 0.200 | 1.352 |  |
|  | **Mixed_Self** |  | 0.055 |  |  |  | 2.016 |  |
|  |  |  |  |  |  |  |  |  |
| **dmPFC** |  | **Mixed_Self** | **2TBs_Self** |  |  |  |  |  |
|  | **2FBs_Self** | 0.202 | 0.871 |  |  | 1.312 | 0.164 |  |
|  | **Mixed_Self** |  | 0.364 |  |  |  | 0.924 |  |
|  |  |  |  |  |  |  |  |  |
| **pmFC** |  | **Mixed_Self** | **2TBs_Self** |  |  |  |  |  |
|  | **2FBs_Self** | 0.019 | 0.527 |  |  | 2.509 | 0.641 |  |
|  | **Mixed_Self** |  | 0.061 |  |  |  | 1.969 |  |
|  |  |  |  |  |  |  |  |  |
| **RIFG** |  | **Mixed_Self** | **2TBs_Self** |  |  |  |  |  |
|  | **2FBs_Self** | 0.013 | 0.327 |  |  | 2.695 | 1.001 |  |
|  | **Mixed_Self** |  | 0.230 |  |  |  | 1.233 |  |
|  |  |  |  |  |  |  |  |  |
| **LIFG** |  | **Mixed_Self** | **2TBs_Self** |  |  |  |  |  |
|  | **2FBs_Self** | 0.039 | 0.863 |  |  | 2.186 | 0.174 |  |
|  | **Mixed_Self** |  | 0.006 |  |  |  | 2.992 |  |

**
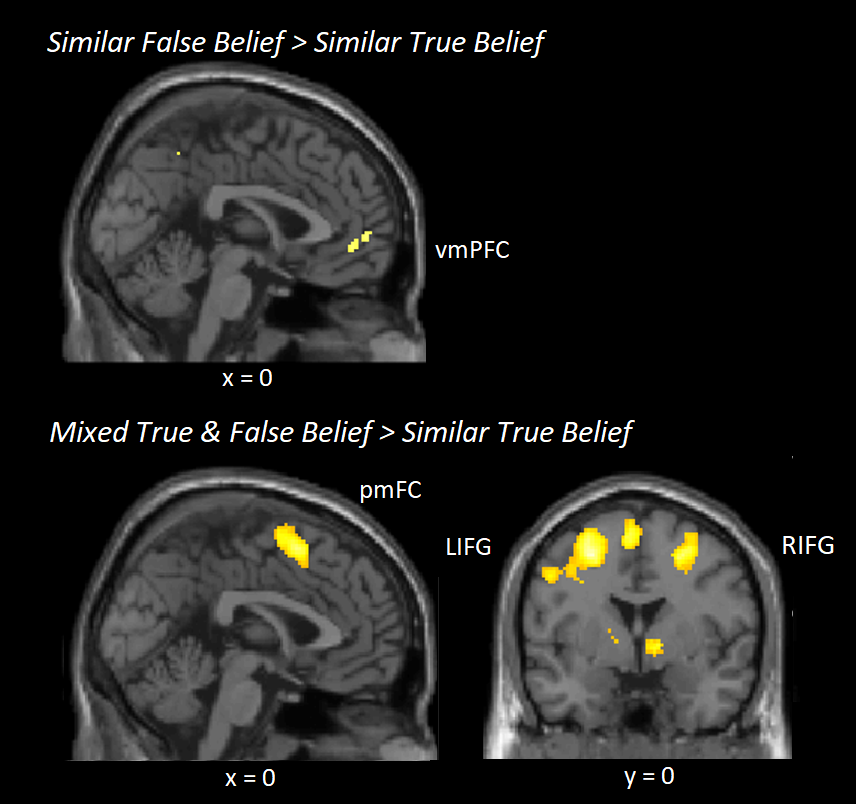
Supplementary Figure 1**: Whole-Brain contrasts during the Story phase showing activation in the ventromedial prefrontal cortex (vmPFC), posterior medial frontal cortex (pmFC), and bilateral inferior frontal gyrus (IFG, L = left, R = right) at *p* < .001 (uncorrected).

**
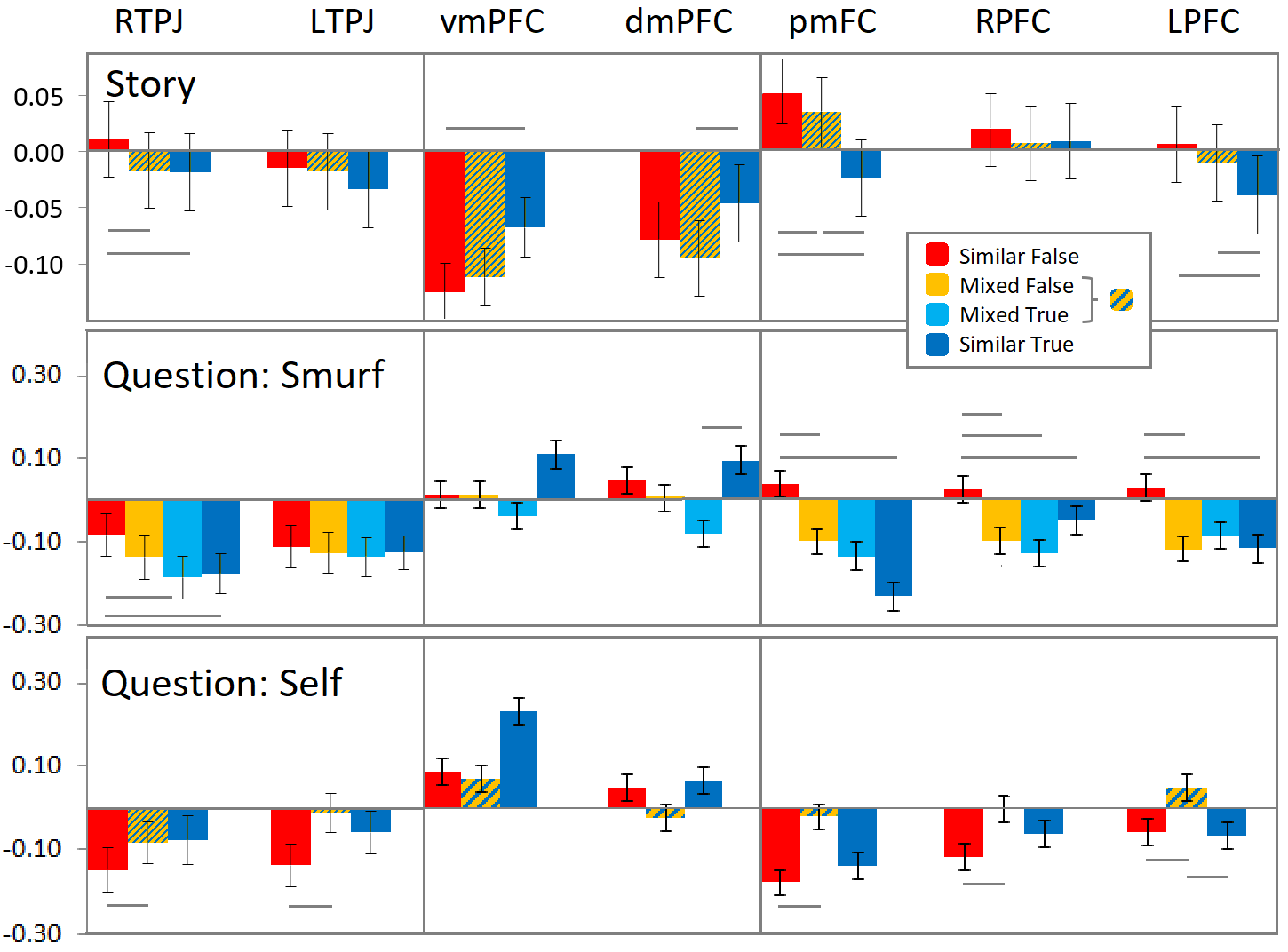
Supplementary Figure 2**: Percentage signal change during Story and Question (Smurf and Self) phase for mentalizing and conflict monitoring ROIs. Horizontal lines denote conditions that differ with *p* ≤ .05 using paired t-tests uncorrected for multiple comparisons. TPJ = temporo-parietal junction (L = left, R = right; MNI coordinates: -55 -65 27; 56 -56 25), medial prefrontal cortex (mPFC; v = ventral and d = dorsal; MNI 0 50 5 and 0 50 35), pmFC = posterior frontal cortex (MNI 0 20 45), inferior frontal gyrus (IFG; L = left, R = right; MNI ± 40 25 20).
